# Syringotropic mycosis fungoides

**DOI:** 10.1002/ski2.353

**Published:** 2024-03-16

**Authors:** Edmond Demoulins, Clémence Berthin

**Affiliations:** ^1^ Department of Dermatology University Hospital Centre Angers Angers France

## Abstract

Brief description of a syringotropic mycosis fungoides.

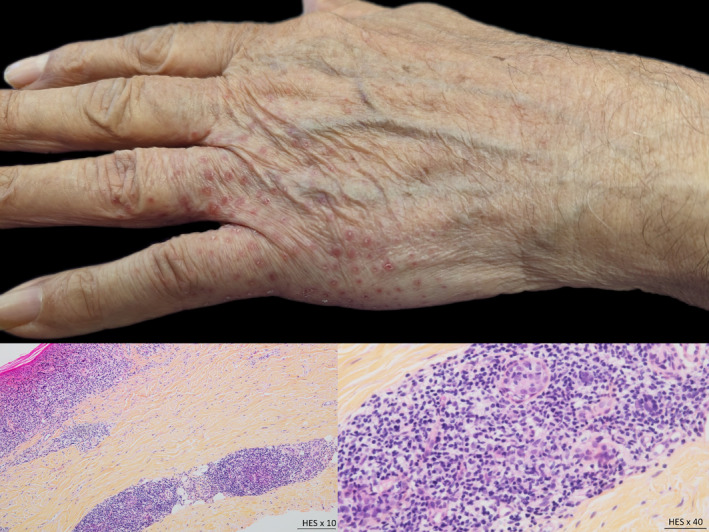

We present the case of a 83‐year‐old patient with symmetrical, 3 months old, lesions of the hands (see Figure 1a).

**FIGURE 1:**
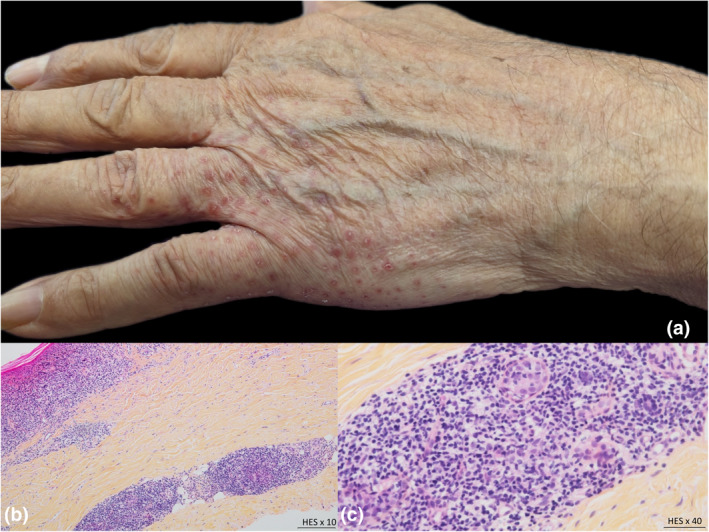
Erythematous scaly lesions on the back of the hand. (a) Clinical photography, (b and c) Microscopic view, x10 and x40 zoom respectively, hematoxylin‐eosin stain.

The histology revealed a syringotropic mycosis fungoides (see Figure 1b,c). This type of mycosis fungoides belongs to the subgroup of follicular mycosis fungoides in the WHO‐EORTC classification of cutaneous lymphomas. It can present a wide variety of clinical symptoms.^1^

Purely pilotropic forms predominantly affect the head and trunk, while syringotropic forms preferentially affect the palms and soles.^2^

In our case, the lesions regressed rapidly following applications of DERMOVAL® (Clobetasol propionate 0.05%), with no recurrence to date.

## CONFLICT OF INTEREST STATEMENT

The authors declare no conflicts of interest.

## AUTHOR CONTRIBUTIONS

**Edmond Demoulins**: Project administration (equal); writing—original draft (equal). **Clémence Berthin**: Validation (equal).

## ETHICS STATEMENT

Not applicable.

## Data Availability

The data underlying this article will be shared on reasonable request to the corresponding author. The data are not publicly available due to privacy or ethical restrictions.
